# Genetic relationship between purebred and crossbred sow longevity

**DOI:** 10.1186/s40104-016-0112-x

**Published:** 2016-09-06

**Authors:** C. E. Abell, R. L. Fernando, T. V. Serenius, M. F. Rothschild, K. A. Gray, K. J. Stalder

**Affiliations:** 1 Department of Animal Science, Iowa State University, Ames, IA 50011-3150 USA; 2 Figen Ltd., PO Box 319, 60101 Seinäjoki, Finland; 3 Smithfield Premium Genetics, 316 W. Charity Rd, Rose Hill, NC 28458 USA

**Keywords:** Binary traits, Sow longevity, Threshold model

## Abstract

**Background:**

The overall breeding objective for a nucleus swine selection program is to improve crossbred commercial performance. Most genetic improvement programs are based on an assumed high degree of positive relationship between purebred performance in a nucleus herd and their relatives’ crossbred performance in a commercial herd. The objective of this study was to examine the relationship between purebred and crossbred sow longevity performance. Sow longevity was defined as a binary trait with a success occurring if a sow remained in the herd for a certain number of parities and including the cumulative number born alive as a measure of reproductive success. Heritabilities, genetic correlations, and phenotypic correlations were estimated using THRGIBBS1F90.

**Results:**

Results indicated little to no genetic correlations between crossbred and purebred reproductive traits. This indicates that selection for longevity or lifetime performance at the nucleus level may not result in improved longevity and lifetime performance at the crossbred level. Early parity performance was highly correlated with lifetime performance indicating that an indicator trait at an early parity could be used to predict lifetime performance. This would allow a sow to have her own record for the selection trait before she has been removed from the herd.

**Conclusions:**

Results from this study aid in quantifying the relationship between purebred and crossbred performance and provide information for genetic companies to consider when developing a selection program where the objective is to improve crossbred sow performance. Utilizing crossbred records in a selection program would be the best way to improve crossbred sow productivity.

## Background

In a commercial swine breeding herd, sow longevity is a contributing factor to the operation’s overall success and profitability. A sow must remain in the herd between 3 and 4 parities to produce enough piglets before the investment in her reaches a positive net present value or “she pays for herself” [1]. Increasing the number of lifetime piglets produced per sow reduces the proportion of the sow’s replacement and development costs that must be recovered by each pig. Because of this, a sow should not be voluntarily culled from the breeding herd as long as she is producing litters with the same number and quality of pigs as the herd average and she does not suffer from welfare issues.

Additionally, removing females in early parities does not allow a swine operation to benefit from the higher performance of sows compared to gilts and down-stream grow-finish performance from pigs produced from parity 1 sows versus performance from pigs produced by older sows (parity 2 and greater). Not only do sows tend to have larger litters compared to gilts [2–4], piglets from sow litters tend to have decreased mortality and improved or superior performance throughout the nursery and grow-finish phases when compared to piglets from gilt litters [5–7]. The increased performance of pigs from sow litters compared to pigs from gilt litters is more valuable to the swine operation than the increased litter size from sows compared to gilts. The performance difference value between the offspring from gilt litters when compared to the offspring from older parity sows will likely dwarf the value of additional parities based on sow performance only [8]. Under maximum genetic gain conditions, a gilt’s genetic advantage is not sufficient to cover the variable costs associated with gilt development until the sow has reached at least parity 7 [9]. Therefore, sows should not be replaced with gilts based on improved genetic potential alone until the sow has reached parity 7 or greater. With slower rates of genetic gain and longer generation intervals, the optimal culling time based solely on genetic potential is the 10th or 11th parity [9]. Because of this, sow reproductive performance should be used to make culling decisions at the commercial level rather than an expectation of improved genetic potential and performance from a replacement gilt.

The overall breeding objective for a swine nucleus selection program is to improve crossbred commercial performance. Most genetic improvement programs are based on an assumed high degree of positive relationship between purebred performance in a nucleus herd and their relatives’ crossbred performance in a commercial herd. In addition to the sows’ genetic makeup differences (i.e. purebred versus crossbred) in nucleus and commercial herds, there are differences in management practices between the two types of herds. Sow longevity is an economically important trait at the commercial level; however, nucleus sows typically produce fewer parities due to the desire to shorten the generation interval in order to increase the rate of genetic improvement. Removing sows at the nucleus level before they have fully expressed their lifetime potential does not allow for direct selection on longevity in the nucleus herd. Since nucleus animals do not have phenotypic records for lifetime measurements, an indicator trait expressed at an earlier parity would be necessary to select for longevity. Few sows remain in a nucleus herd after the 2nd parity due to culling for genetic potential. If 2nd parity reproductive performance is indicative of lifetime performance in commercial animals and if a genetic correlation exists between purebred and crossbred performance, selection on nucleus animals after only 2 parities could have a positive impact on improving sow longevity and lifetime production in commercial herds.

The objective of this study was to examine the relationship between purebred and crossbred lifetime reproductive performance. The traits of interest were whether or not a sow remained in the herd up to a certain number of parities and the total number born alive for a sow across parities, or cumulative born alive. Defining the relationship between purebred and crossbred sow longevity will allow genetic suppliers to improve selection methods for increased longevity at the commercial level.

## Methods

Five nucleus herds and 2 commercial herds were used for this analysis. All 7 herds were owned by the same company and operated using the same management protocols. The commercial herds were part of the downstream production pyramid from the nucleus herds. Removal records for 11,506 purebred sows and 12,897 crossbred sows were evaluated for this study. Purebred sows were from a Landrace pure line, and the crossbred sows were F1 offspring from a cross between the Landrace pure line and a Large White pure line. All crossbred animals were produced using a Large White female and Landrace male. There were 1,039 and 213 sires for the purebred and crossbred sows, respectively. Of the 213 sires used at the crossbred level, 205 had dams with records at the purebred level, providing genetic ties between the purebred and crossbred herds. No sires were used at both the nucleus and commercial herds. Purebred records were from sows first farrowing in February 1993 through May 2011. Crossbred sows first farrowed in November 2004 to May 2011. Sows that were active or that were continuing to produce (not yet completed their lifetime record) in the herd were included in the analysis, but not treated as censored data. Previous work has shown that if the number of completed records overwhelms the number of censored records, there is little re-ranking among the breeding value estimations [10]. Pedigree information for at least three generations was known for each individual with performance records.

Sow longevity traits were defined based on the sow’s removal parity. At the nucleus level, sows were considered successful if they completed parity 2 (farrowed and weaned their second litter). This trait is called PL2. At the commercial level, four degrees of success (Parity 2, Parity 3, Parity 4, and Parity 5) were analyzed as separate traits. These traits are called CL2, CL3, CL4, and CL5, respectively. Additionally, cumulative number born alive traits were defined for purebred and crossbred sows. For purebred sows, cumulative number born alive up to parity two (PNBA2) and for a lifetime (PNBALF) were defined. For crossbred sows, cumulative number born alive up to parity 2 (CNBA2), parity 3 (CNBA3), parity 4 (CNBA4), parity 5 (CNBA5) and for a lifetime (CNBALF) were defined. Descriptive statistics for the cumulative and lifetime born alive traits are shown in Table 1. The heritabilities for each trait and genetic correlations between the nucleus and commercial level successes were estimated.

**Table 1:** Descriptive statistics for cumulative born alive traits in purebred and crossbred sows^a^

Trait^b^	Mean ± SD	Minimum	Maximum
PNBA2	17.4 ± 7.4	0	37
PNBALF	27.5 ± 19.5	0	132
CNBA2	21.6 ± 6.8	0	40
CNBA3	30.6 ± 12.2	0	59
CNBA4	38.1 ± 17.9	0	81
CNBA5	44.5 ± 22.9	0	93
CNBALF	50.2 ± 30.0	0	146

^a^Purebred sows were from a Landrace pure line, and the crossbred sows were F1 offspring from a cross between a Landrace pure line and a Large White pure line

^b^PNBA2 and PNBALF represent the cumulative number born alive for purebred sows at parity 2 and for their lifetime. CNBA2, CNBA3, CNBA4, CNBA5, and CNBALF represent the cumulative number born alive for purebred sows at parity 2, parity 3, parity 4, parity 5, and for their lifetime

All traits were analyzed using THRGIBBS1F90 [11] with 100,000 iterations. Of the 100,000 iterations, 10,000 were used for burn-in and 90,000 for estimations. The traits Pl2, Cl2, CL3, CL4, and CL5 were analyzed as binary traits, and PNBA2, PNBALF, CNBA3, CNBA3, CNBA4, CNBA5, and CNBALF were analyzed as linear traits. The following model was used to analyze all traits:

$$ \boldsymbol{y}=\mathbf{X}\upmu +\mathbf{W}\mathbf{f}+\mathbf{Z}\mathbf{u}+\mathbf{e} $$

where **y** is the vector of observations, μ is the mean, **f** is the vector of contemporary group effects, **u** is the additive genetic animal effects, **e** is the vector of residual effects, and **X**, **W**, and **Z** are known incidence matrices. Herd, year, and month of last farrowing were used to define the contemporary groups. Contemporary group was fitted as a fixed effect for all traits analyzed. All models included a random animal effect. Bivariate analyses were used to estimate genetic and phenotypic correlations, and univariate analyses were used to estimate heritabilities.

Heritability was defined as

$$ {h}^2=\frac{\sigma_g^2}{\sigma_g^2+{\sigma}_e^2} $$

where *σ*_*g*_^2^ is the additive genetic variance, and *σ*_*e*_^2^ is the residual variance. The genetic correlation between traits *x* and *y* was calculated as

$$ {r}_{g_{x,y}}=\frac{\sigma_{g_{x,y}}}{\sqrt{\sigma_{g_x}^2\times {\sigma}_{g_y}^2}} $$

where ${\sigma}_{g_{x,y}}$ is the genetic covariance between *x* and *y*, ${\sigma}_{g_x}^2$ is the genetic variance of *x*, and ${\sigma}_{g_y}^2$ is the genetic variance of *y*. The phenotypic correlation between traits *x* and *y* was calculated as

$$ {r}_{p_{x,y}}=\frac{\sigma_{g_{x,y}}+{\sigma}_{e_{x,y}}}{\sqrt{\left({\sigma}_{g_x}^2+{\sigma}_{e_x}^2\right)\times \left({\sigma}_{g_y}^2+{\sigma}_{e_x}^2\right)}} $$

where ${\sigma}_{g_{x,y}}$, ${\sigma}_{g_x}^2$, and ${\sigma}_{g_y}^2$ are as defined above and ${\sigma}_{e_{x,y}}$ is the residual correlation between *x* and *y*, ${\sigma}_{e_x}^2$ is the residual variance of *x*, and ${\sigma}_{e_y}^2$ is the residual variance of *y*. The estimates were calculated for each sample.

Using the genetic variance estimate for PNBALF, the relative economic importance for lifetime pigs produced compared to other reproductive traits was determined. The genetic variance along with the heritability indicates how much genetic progress can be made in a trait. The economic value of a one unit improvement in the trait can be used to determine the relative economic performance of the potential genetic progress compared with other traits in the index. The other traits included in the analysis were number born alive (NBA), number weaned (NW), litter weaning weight (LW), litter birth weight (LBW), and wean to estrus interval (WTE). The genetic standard deviations for these traits were obtained from commercial seedstock suppliers. The economic weights were $27.93 for a pig born alive, $38.57 for weaned pig, $0.45 per pound of live weight, and $1.90 cost for a nonproductive day [12]. The standardized economic weight was calculated by multiplying the economic value by the genetic standard deviation. The relative economic value for each trait was calculated as the standardized economic value for the trait divided by the sum total of the standardized economic values [13].

## Results

The removal parity distributions for the purebred and crossbred sows are in Table 2. The average removal parity for purebred sows was 2.5, while the average removal parity for the crossbred sows was 3.8. It is clear that crossbred sows remained in the herd for more parities on average compared to purebred sows. This would be expected as nucleus herds must turn over the sow herd as rapidly as possible in order to maximize rate of genetic gain.

**Table 2:** Removal parity distribution for crossbred and purebred sows^a^

Removal parity	Crossbred sows, %	Purebred sows, %
1	20.8	36.8
2	14.1	20.3
3	10.9	17.1
4	13.9	14.3
5	13.0	8.8
6	14.6	1.9
7	9.1	0.6
8	3.3	0.3
9	0.3	0.1

^a^Purebred sows were from a Landrace pure line, and the crossbred sows were F1 offspring from a cross between a Landrace pure line and a Large White pure line

The heritability, genetic correlation, and phenotypic correlation estimates are presented in Table 3. Heritabilities for the binary traits were high in magnitude with varying standard deviations. The high heritability for PL2 is not unexpected since selection decisions at the nucleus are made based on the genetic potential of a sow, which would allow for sows from the same families to be selected to remain in the herd for an additional parity. The genetic correlations between the purebred longevity trait and crossbred longevity traits were not significantly different from 0, suggesting that a sow remaining in the nucleus herd for at least 2 parities is not indicative of her offspring’s longevity at the commercial level. Heritability estimates for all of the crossbred traits were similar and do not suggest that any longevity definition would be better to incorporate into a genetic program compared to the other definitions based on heritability alone. The genetic correlation between CL2 and CNBALF was high (0.83) indicating that a commercial sow’s ability to produce two parities is indicative of her lifetime reproduction performance.

**Table 3:** Heritability (on diagonal), genetic correlation (above diagonal), and phenotypic correlation (below diagonal) estimates (SD) between sow reproductive performance and longevity traits using an animal model^a^

	PL2^b^	CL2	CL3	CL4	CL5	PNBA2	PNBALF	CNBA2	CNBA3	CNBA4	CNBA5	CNBALF
PL2	0.81 (0.03)	0.38 (0.30)	0.39 (0.29)	0.38 (0.30)	0.40 (0.29)	0.98 (0.05)	>0.99 (<0.01)	0.18 (0.09)	0.19 (0.08)	0.18 (0.08)	0.17 (0.08)	0.17 (0.07)
CL2	0.00 (0.02)	0.98 (0.02)	0.96 (0.03)	0.80 (0.11)	0.63 (0.24)	0.05 (0.03)	0.02 (0.03)	0.18 (0.35)	0.96 (0.01)	0.93 (0.01)	0.89 (0.02)	0.83 (0.03)
CL3	0.01 (0.03)	0.93 (0.04)	0.90 (0.29)	0.93 (0.08)	0.71 (0.28)	0.05 (0.03)	0.02 (0.03)	0.78 (0.03)	0.95 (0.01)	0.96 (0.01)	0.96 (0.01)	0.92 (0.02)
CL4	0.01 (0.02)	0.67 (0.14)	0.81 (0.14)	0.82 (0.38)	0.84 (0.15)	0.05 (0.03)	0.02 (0.03)	0.68 (0.05)	0.90 (0.02)	0.95 (0.01)	0.97 (0.01)	0.96 (0.01)
CL5	0.00 (0.03)	0.46 (0.21)	0.56 (0.25)	0.67 (0.19)	0.81 (0.39)	0.05 (0.03)	0.02 (0.03)	0.61 (0.07)	0.82 (0.03)	0.89 (0.02)	0.95 (0.01)	0.96 (0.01)
PNBA2	0.87 (0.04)	0.02 (0.02)	0.03 (0.02)	0.03 (0.02)	0.03 (0.02)	0.27 (0.02)	0.94 (0.01)	0.30 (0.10)	0.31 (0.08)	0.31 (0.07)	0.29 (0.07)	0.29 (0.07)
PNBALF	0.98 (0.01)	0.03 (0.03)	0.04 (0.03)	0.04 (0.02)	0.04 (0.03)	0.74 (<0.01)	0.52 (0.02)	0.23 (0.07)	0.25 (0.06)	0.27 (0.06)	0.27 (0.06)	0.28 (0.06)
CNBA2	0.11 (0.05)	0.18 (0.32)	0.42 (0.02)	0.24 (0.03)	0.14 (0.02)	0.10 (0.03)	0.11 (0.04)	0.46 (0.03)	0.94 (0.01)	0.88 (0.02)	0.81 (0.02)	0.74 (0.03)
CNBA3	0.14 (0.06)	0.85 (0.02)	0.78 (0.01)	0.53 (0.02)	0.34 (0.02)	0.14 (0.04)	0.16 (0.04)	0.86 (<0.01)	0.78 (0.03)	0.99 (<0.01)	0.96 (0.01)	0.92 (0.01)
CNBA4	0.15 (0.07)	0.86 (0.02)	0.79 (0.04)	0.79 (0.04)	0.55 (0.02)	0.16 (0.04)	0.19 (0.04)	0.72 (<0.01)	0.93 (<0.01)	0.98 (0.01)	0.99 (<0.01)	0.97 (<0.01)
CNBA5	0.14 (0.07)	0.83 (0.02)	0.89 (0.01)	0.88 (0.05)	0.79 (0.02)	0.15 (0.04)	0.19 (0.04)	0.62 (0.01)	0.83 (<0.01)	0.95 (<0.01)	0.99 (<0.01)	0.99 (<0.01)
CNBALF	0.14 (0.06)	0.73 (0.02)	0.83 (0.02)	0.86 (0.01)	0.89 (0.01)	0.15 (0.03)	0.20 (0.04)	0.49 (0.01)	0.68 (<0.01)	0.81 (<0.01)	0.91 (<0.01)	0.99 (<0.01)

^a^Purebred sows were from a Landrace pure line, and the crossbred sows were F1 offspring from a cross between a Landrace pure line and a Large White pure line

^b^Trait abbreviations starting with P indicates purebred records and C indicates crossbred records. PL2, CL2, CL3, CL4, and CL5 are binary traits. For PL2, a success is considered to be a sow that remains in the nucleus herd long enough to farrow 2 litters. For CL2, CL3, CL4, and CL5, a success is considered to be a sow that remains in the commercial herd long enough to farrow 2, 3, 4, and 5 litters, respectively. PNBA2 and PNBALF represent the cumulative number born alive for purebred sows at parity 2 and for their lifetime. CNBA2, CNBA3, CNBA4, CNBA5, and CNBALF represent the cumulative number born alive for purebred sows at parity 2, parity 3, parity 4, parity 5, and for their lifetime. The estimates were calculated using THRGIBBS1F90 and the model described in the text

The genetic correlations between the purebred and crossbred cumulative and lifetime born alive traits were not significantly different from 0. However, the genetic correlations between all crossbred cumulative born alive traits were high (>0.70), suggesting that early parity performance is indicative of a sow’s lifetime performance in a commercial herd.

The relative economic weights for reproductive traits are shown in Table 4. Based on these results, it is clear that lifetime born alive has the greatest relative economic importance compared to the other reproductive traits. This suggests that genetic companies should have more selection pressure on sow lifetime productivity compared to other reproductive traits in their selection programs. Number born alive at a single litter is the second most relevant trait.

**Table 4:** Relative economic emphasis of swine reproductive traits

Trait^c^	Economic Value per Trait Unit (v_i_)	Genetic Standard Deviation (σ_g_)	Standardized Economic Weight (E_i_)^a^	Relative Emphasis (E)^b^
NBA	$27.93	0.70	19.55	0.049
NW	$38.57	0.28	10.91	0.027
LW	$0.45	8.07	3.63	0.009
WTE	$1.90	1.05	2.00	0.005
LBW	$0.45	2.92	1.32	0.003
NBALF	$27.93	12.90	360.29	0.906

^a^E_i_ = v_i_ * σ_g_

^b^E = E_i_ / Σ_6_ (E_i_)

^c^ *NBA* Number born alive, *NW* number weaned, *LW* litter weaning weight, *WTE* wean to estrus interval, *LBW* litter birth weight, *NBALF* lifetime born alive

## Discussion

Improving crossbred performance is the goal for most swine breeders. However, most swine genetic companies make selection decisions based on purebred performance records though genetic correlations between purebred and crossbred performance traits are not 1, indicating that the relationship is not perfect [14–16]. While the magnitude of the genetic correlation estimates between the cumulative and lifetime born alive traits in this study were similar to correlations estimates between purebred and crossbred traits previously reported, the genetic correlations between longevity traits in the current study were lower in magnitude compared to other studies [14–16]. The population structure in the current analysis could have prevented the detection of a genetic correlation. A structure where sires were used in both the purebred and crossbred herds would be more ideal to estimate genetic correlations; however, ideal data structures are not always available when analyzing field data. Additionally, the methods utilized in the study may not be sufficient in detecting a genetic association between the binary traits due to the dataset’s population structure.

The greater genetic correlation estimates between crossbred and purebred longevity found in previous studies [14–16] compared to the estimates from this study could be a result of the traits considered in the previous studies being easier to define and quantify. Longevity is based on culling criteria which can vary between nucleus and commercial levels and from farm-to-farm. Many criteria used to make removal decisions are subjective. The genetic correlations found in this study may indicate that longevity is a different trait at the purebred and crossbred levels due to the culling criteria used at each production level. Therefore, there may be few genes that have large associations with the different longevity definitions.

Previously reported literature estimates have shown that heritabilities and genetic correlations between traits in a selection index vary between crossbred and purebred populations [17–19]. The same is true for the results from this study. Since coefficients for selection indices are derived based on genetic correlations and trait heritabilities, these genetic parameter differences among purebred and crossbred populations can impact the selection index coefficients. If index weights are incorrect, selection would not be optimized, and rate of genetic gain for the overall index would decrease. Since the breeding objective of most swine genetics companies is to improve crossbred performance, genetic gain would be optimized if crossbred records were used to make selection decisions at the purebred level.

In this study, over 40 % of the purebred sows remained in the herd for greater than 2 parities. This number is inflated from what would be expected in some companies if a nucleus herd was trying to maximize genetic progress as quickly as possible. Sows may remain in a nucleus herd due to a reduction in available gilts to enter the herd or fewer lower parity sows. This decreased supply of gilts and low parity sows could be a result from a health challenge to the system. Keeping animals in the herd that would have otherwise been removed could impact the heritability associated with sow longevity. Typically, a female is retained in a nucleus herd for additional parities when the older female has an index value greater than the index value of potential replacement gilts. Changing the selection criteria would result in changing the longevity definition, which affects the genetic parameters associated with the trait. For example, sows that survive a health challenge may not have the same characteristics as sows that have high reproductive performance in a herd with a high health status.

For this analysis, there were approximately equal numbers of purebred and crossbred sows due to the longer data collection period for purebred sows. Since this analysis presented evidence that there is little to no genetic correlation between purebred and crossbred longevity measures, genetic companies should make use of maternal records from their crossbred sow population through the development of a commercial test herd or develop relationships with customers willing to keep meticulous records. In either case, these records can be used to augment the purebred records for more lowly heritable traits to improve accuracy and speed genetic progress.

Heritability estimates of 0.03 and 0.08 for lifetime born alive in crossbred sows, which are smaller than the estimate found in this study, have been reported previously [20]. Additionally, a high genetic correlation (0.61–0.93) between lifetime pigs born alive and stayability from parity 1 to parity 2 and 3 for crossbred sows has been reported [20]. This suggests that lifetime pigs born alive is a good indicator of a longevity. Cumulative born alive is a combination of length of productive life and litter size. Using the cumulative born alive estimates would be different from selecting for litter size alone, since sows producing for fewer parities would be penalized in the cumulative born alive trait. Thus, improving cumulative born alive would improve sow lifetime productivity rather than productivity at a single parity.

## Conclusions

Because of the large relative emphasis that should be placed on lifetime born alive, genetic companies must focus on adding sow lifetime production to a selection program. One way to do this would be to implement a commercial test herd. With the commercial test herd, genetic companies could collect data from crossbred animals to be used in the genetic evaluations. Increasing the amount of data collected on crossbred females would allow genetic companies to more accurately select to improve crossbred performance in commercial herds.
